# Assessing Positivity and Circulating Levels of NS1 in Samples from a 2012 Dengue Outbreak in Rio de Janeiro, Brazil

**DOI:** 10.1371/journal.pone.0113634

**Published:** 2014-11-20

**Authors:** Diego Allonso, Marcelo D. F. Meneses, Carlos A. Fernandes, Davis F. Ferreira, Ronaldo Mohana-Borges

**Affiliations:** 1 Instituto de Biofísica Carlos Chagas Filho, Universidade Federal do Rio de Janeiro, Rio de Janeiro, RJ, Brazil; 2 Instituto de Microbiologia Paulo de Góes, Universidade Federal do Rio de Janeiro, Rio de Janeiro, RJ, Brazil; 3 Laboratorio Central Noel Nütels (LACEN), Rio de Janeiro, RJ, Brazil; Centro de Pesquisas René Rachou, Brazil

## Abstract

Dengue virus (DENV) represents a major threat to public health worldwide. Early DENV diagnosis should not only detect the infection but also identify patients with a higher likelihood to develop severe cases. Previous studies have suggested the potential for NS1 to serve as a viral marker for dengue severity. However, further studies using different sera panels are required to confirm this hypothesis. In this context, we developed a lab-based ELISA to detect and quantitate NS1 protein from the four DENV serotypes and from primary and secondary cases. This approach was used to calculate the circulating NS1 concentration in positive samples. We also tested the NS1 positivity of DENV-positive samples according to the Platelia Dengue NS1 Ag assay. A total of 128 samples were positive for DENV infection and were classified according to the WHO guidelines. The overall NS1 positivity was 68% according to the Platelia assay, whereas all samples were NS1-positive when analyzed with our lab-based ELISA. Fifty-four samples were positive by PCR, revealing a co-circulation of DENV1 and DENV4, and the NS1 positivity for DENV4 samples was lower than that for DENV1. The circulating NS1 concentration ranged from 7 to 284 ng/mL. Our results support previous data indicating the low efficiency of the Platelia assay to detect DENV4 infection. Moreover, this work is the first to analyze NS1 antigenemia using retrospective samples from a Brazilian outbreak.

## Introduction

Classified by the World Health Organization (WHO) as one of the most prevalent arthropod-borne viruses in the world, dengue virus (DENV) constitutes a serious public health problem. It is estimated that at least 40% of the world's population (2.5 billion people) live in areas where DENV is endemic. This infection results in more than 50 million cases annually and tens of thousands of deaths [1]. Dengue disease presents a wide range of clinical manifestations commonly described as undifferentiated febrile illness, known as dengue fever (DF), although the disease may evolve into severe hemorrhage, which is potentially fatal and classically termed dengue hemorrhagic fever/dengue shock syndrome (DHF/DSS) [2]. In 2009, the WHO adopted a new classification of dengue based on the clinical manifestation spectra, including dengue without or with warning signs and severe dengue [3]. According to this classification, patients have dengue when they exhibit fever and two of the following symptoms: nausea, rash, aches and pains, leucopenia and a positive tourniquet test. These patients are sent home because they are not likely to develop severe dengue. However, if patients exhibit abdominal pain, persisting vomiting, fluid accumulation, mucosal bleeding, lethargy and/or liver enlargement, besides the abovementioned symptoms, they present dengue with warning signs and require strict observation and medical intervention. These patients may evolve to severe dengue characterized by severe plasma leakage, bleeding and organ impairment. This classification is believed to facilitate initial clinical care as well as clinical management and surveillance, with focused attention on the latter two cases, which are potentially dangerous.

Although initial dengue classification is essentially clinical, it is imperative to obtain laboratory confirmation. Until the 2000's, dengue diagnosis was commonly performed during the convalescent phase via the detection of specific antibodies against DENV (IgM and/or IgG). After the discovery that the non-structural protein 1 (NS1) circulates in the serum of infected patients during the acute phase of the disease [4], [5], a large number of kits based on circulating NS1 detection were developed and commercialized. These tools represent important progress in early dengue detection, but their performance varies according to the manufacturer, the infecting serotype and the immune status of the patient (primary or secondary infection) [2], [6]–[8]. Thus, it remains important to improve current methodologies to differentiate dengue from other febrile illnesses and to perform epidemiological studies.

Ideal early dengue diagnosis should not only detect the infection but also identify patients with a higher likelihood to develop severe cases. Various endogenous molecules, including altered cytokine profiles, have been described as a promising viral marker for disease progression [9]–[16]. However, these molecules are not specific to dengue infection, are generally not stable in solution (serum samples) and present a restricted detection window, thereby limiting their potential usefulness. A previous prospective study in Thai children with DENV2 reported that the circulating NS1 concentration was significantly higher in those experiencing DHF in the first 72 h of illness, and these authors concluded that NS1 may represent a marker for dengue severity [17]. Another report also using samples from Thai children confirmed an augmentation in NS1 levels in DHF patients when compared with DF patients during the very early infection period [18]. In contrast, Watanabe and colleagues, using a mouse model, demonstrated that the magnitude of NS1 secretion depended on the infecting serotype and did not correlate with severity [19]. A recent study performed in Mexico also showed that circulating NS1 levels were higher for patients experiencing DHF than those with DF when the infection was caused by DENV1 [20]. Conversely, no significant difference was observed in NS1 levels in DENV2 infection. Thus, despite great interest in the search for new markers of disease evolution, there are few reports describing the potential of NS1 for this role.

In the present study, we conducted a qualitative and quantitative analysis of NS1 antigenemia in serum samples from DENV-infected patients collected during a 2012 outbreak that occurred in Rio de Janeiro, Brazil. Specifically, we developed a lab-based ELISA that could detect and quantitate the NS1 antigen of all four DENV serotypes, and we then compared the NS1 positivity between our lab-based system and the Platelia Dengue NS1 Ag assay from Bio-Rad. After determined the circulating NS1 concentration in these serum samples, we did not observe any difference between non-severe and severe cases of dengue. Furthermore, our study is the first to analyze NS1 antigenemia using retrospective samples from a Brazilian outbreak, which is the country most affected by DENV in Latin America, based on the new WHO classification of dengue severity.

## Materials and Methods

### Ethics Statement

This study was reviewed and approved by the Research Ethics Committee for Clinical Studies of the University Hospital Clementino Fraga Filho of the Federal University of Rio de Janeiro (UFRJ) and registered in the Brazilian Human Ethics Committee (Plataforma Brasil, CAAE: 00534012.6.0000.5257, expiration date: June 3^rd^, 2017). Informed consent was not obtained as patient records/information was anonymized and de-identified prior to analysis.

The Ethics Committee for Animal Use at the Health Science Center of UFRJ approved the protocols for the production of polyclonal antibodies (registration numbers: IBCCF146 and IBCCF147).

### Sera panel

All DENV-positive samples used in the present study were collected during the 2012 dengue outbreak that occurred in Rio de Janeiro, Brazil. These samples were obtained from Noel Nütels Central Laboratory of Rio de Janeiro (LACEN-RJ) sera bank. This laboratory is a government center for epidemiological study of several infectious diseases and is responsible for epidemiological bulletins and pest control strategies. LACEN receives samples from different cities within the state of Rio de Janeiro and, in probable cases of dengue, specific assays are performed, such as dengue-specific IgM and IgG detection (also used to distinguish between primary and secondary infections), serotype-specific PCR and NS1 detection using the Platelia Dengue NS1 Ag assay. The samples from apparently health donors were obtained from a personal collection at the Department of Virology, Microbiology Institute Paulo de Góes, Federal University of Rio de Janeiro (UFRJ), Brazil.

### Serotype-specific PCR, IgM/IgG detection and NS1 detection

Serotype-specific PCR was performed according to Lanciotti *et al.* protocol [21]. Dengue IgM capture ELISA and Dengue IgG ELISA test from Panbio were used to detected dengue specific IgM and IgG, respectively, and Platelia Dengue NS1 Ag assay was used to detect NS1 protein. All assays were performed according to manufacturer's instructions.

### Expression and purification of recombinant DENV NS1 protein

The full-length *ns1* gene from the four serotypes (the strains of each serotype are summarized in Table 1) were inserted into the pET23b plasmid by GenScript (USA). A DNA sequence coding for six histidine amino acids was added at the 3′ end of the *ns1* gene in all constructions. The recombinant plasmids were inserted into *E. coli* BL21(λDE3)pLysS cells, and the expression and purification steps were performed as described previously [22]. Unfolded recombinant NS1 was subjected to a slow 20-fold dilution with 50 mM Tris.HCl pH 8, 100 mM NaCl, 1 mM β-mercaptoethanol and 0.1% (w/v) ASB-14, followed by final dialysis in 50 mM Tris-HCl (pH 8), 100 mM NaCl, 1 mM DTT and 0.05% ASB-14. After refolding, the proteins were concentrated using a Centriprep YM-10 system (Millipore Corporation, USA).

**Table 1:** DENV strains used as template for production of recombinant NS1 protein.

Serotype	Strain	Reference
DENV1	BR/90	GenBank: AF226685.2
DENV2	New Guinea C	GenBank: FJ390389.1
DENV3	BR/74886/02	GenBank: AY679147.1
DENV4	V1156/2007	GenBank: GQ868645.1

### Production of anti-NS1 polyclonal antibodies

Briefly, five-week-old male Balb/c mice were intraperitoneally immunized with 20 µg of rNS1 per animal in complete Freund's adjuvant (Sigma, USA) followed by four injections of the protein in incomplete Freund's adjuvant (Sigma, USA), which were administered two weeks apart. Two weeks after the final immunization, the animals were euthanized and bled by total cardiac puncture. A three-month-old isogenic male rabbit was first subcutaneously immunized with 1 mg of rNS1 in complete Freund's adjuvant followed by two subcutaneous injections of rNS1 in incomplete Freund's adjuvant, which were administered two weeks apart. One week after the final immunization, approximately 20 mL of blood was obtained by ear bleeding. The serum samples were purified by affinity chromatography using protein G columns according to the manufacturer's instructions (GE Healthcare, USA) and stored at −20°C for subsequent analysis. All antibodies reached titer higher than 1∶256,000. Each set of animals received rNS1 of a different serotype.

### DENV NS1 capture ELISA

The wells of a 96-well microtiter plate (Nalge Nunc, USA) were coated at 4°C overnight with 10 µg/mL of protein G-purified anti-rNS1 mouse polyclonal antibodies specific for DENV2 and DENV4 at a 1∶1 ratio in PBS buffer (8.06 mM sodium phosphate, 1.94 mM potassium phosphate, 2.7 mM KCl, and 137 mM NaCl; pH 7.4). The plates were blocked for 1 h at 37°C with 1% BSA in PBST (0.05% Tween 20 in PBS) and then washed five times with PBST buffer. This step was performed after each period of incubation. The wells were separately incubated with serial dilutions of rNS1 for all serotypes, ranging from 15.6 ng/mL to 1 µg/mL, or with patient sera diluted 1∶5 in PBS for 1 h at 37°C. Subsequently, the wells were incubated with protein G-purified rabbit anti-rNS1 polyclonal antibodies specific for DENV2 and DENV4 diluted 1∶6,000 in PBS buffer containing 1% skim milk for 1 h at 37°C, followed by incubation for 1 h at the same temperature with anti-IgG rabbit antibody conjugated to horseradish peroxidase (Promega, USA). After 20 min at room temperature, the reactions were developed with OPD (Sigma Aldrich, USA) and H_2_O_2_ as the substrates and 12.5% H_2_SO_4_ as the quencher. Reactions were monitored by measuring the absorbance at 490 nm. The standard curve calculation was performed using the mean mass value of the serial dilution of the rNS1 protein against its respective optical density (O.D.) measurement. The O.D. measurements were normalized using the mean value of the negative control replicates. Each sample was tested just once.

### Statistical analysis

All quantitative data were summarized by medians and interquartile range (5–95%). Comparisons were carried out using the non-parametric Mann-Whitney U test. All calculations were performed using GraphPad Prism version 5.00 for Windows (GraphPad Software, San Diego California USA), and *p* values below 0.05 were considered statistically significant.

## Results

### Sample characteristics

Serum samples from patients in different cities of the state of Rio de Janeiro, Brazil, who had been clinically diagnosed with dengue infection, were sent to LACEN-RJ for serological dengue confirmation. These samples were then subjected to the following tests: PCR analysis to identify the DENV serotype, detection of NS1 antigen using the Platelia Dengue NS1 Ag assay and determination of dengue-specific IgM/IgG levels to distinguish between primary and secondary infections. The samples showing negative results for these three assays were excluded from the final sera panel. In total, 128 samples were used in this work and were classified according to the patients' clinical symptoms, according to the new WHO guideline recommendations [3], as follows: dengue (n = 97), dengue with warning signs (n = 21) and severe dengue (n = 10) (Table 2). The symptoms reported by the physicians are summarized in Table 2. In contrast to what was observed in dengue outbreaks in Asia, adults were still the most affected individuals in Brazil, as observed by the overall median age of 26 years (ranging from 0 to 89 years). The median number of days after symptom onset was 5 (1–9) for the dengue and dengue with warning signs groups and 6 (4–9) for the severe dengue group, considering day 1 as the day of fever onset. It is worth noting that the date of initial symptom onset was provided by the patients and thus may not precisely correspond to the beginning of the illness. The largest number of patients with non-severe dengue (dengue and dengue with warning signs) exhibited a primary infection, 54.2% (64 out of 118), whereas 70% (7 out of 10) of patients with severe dengue represented secondary cases (Table 2). The overall fatality rate was 2.4% (3/128), and all of these patients belonged to severe dengue group, whose fatality rate was 30%.

**Table 2:** Demographic, clinical symptoms and serological status of dengue-positive samples.

	Case classification			
	Dengue (n = 97)	Dengue with warning signs (n = 21)	Severe Dengue (n = 10)	Total (n = 128)
**Demographics**				
Median of age, years (range)	29 (0–77)	20 (6–82)	41 (11–89)	26 (0–89)
Male: female ratio	1.02	1.21	2	1.01
Median of day of illness (range*)	5 (1–9)	5 (2–9)	6 (4–9)	5 (1–9)
**Clinical symptoms**				
Fever	87 (89.6%)	21 (100%)	8 (80%)	116 (90.6%)
Retro-orbital pain	40 (41.2%)	12 (54.6%)	1 (10%)	53 (41.4%)
Myalgia	52 (53.6%)	15 (72.7%)	3 (30%)	70 (54.6%)
Arthralgia	33 (34%)	12 (54.6%)	3 (30%)	48 (37.5%)
Prostration	46 (47.4%)	12 (54.6%)	4 (40%)	62 (48.4%)
Nausea	26 (26.8%)	13 (59%)	2 (20%)	41 (32%)
Vomiting	0 (0%)	19 (86.4%)	3 (30%)	22 (17.1%)
Rash	8 (8.3%)	1 (4.5%)	0 (0%)	9 (7%)
Diarrhea	10 (10.3%)	9 (40.9%)	3 (30%)	22 (17.1%)
Anorexia	10 (10.3%)	2 (9%)	0 (0%)	12 (9.4%)
Prurience	1 (1%)	11 (52.3%)	1 (10%)	13 (10.1%)
Abdominal pain	0 (0%)	2 (9%)	1 (10%)	3 (2.3%)
Bleeding/hemorrhagia	0 (0%)	0 (0%)	3 (30%)	3 (2.3%)
Notspecified	10 (10.3%)	0 (0%)	1 (10%)	11(8.6%)
**Serological status**				
Primary	51 (52.5%)	13 (61.9%)	3 (30%)	67 (52.3%)
Secondary	46 (47.5%)	8 (38.1%)	7 (70%)	61 (47.7%)
**Fatality**	0 (0%)	0 (0%)	3 (30%)	3 (2.4%)

*The first day after symptoms onset was considered as day 1.

### Evaluation of NS1 positivity

**Figure 1:**
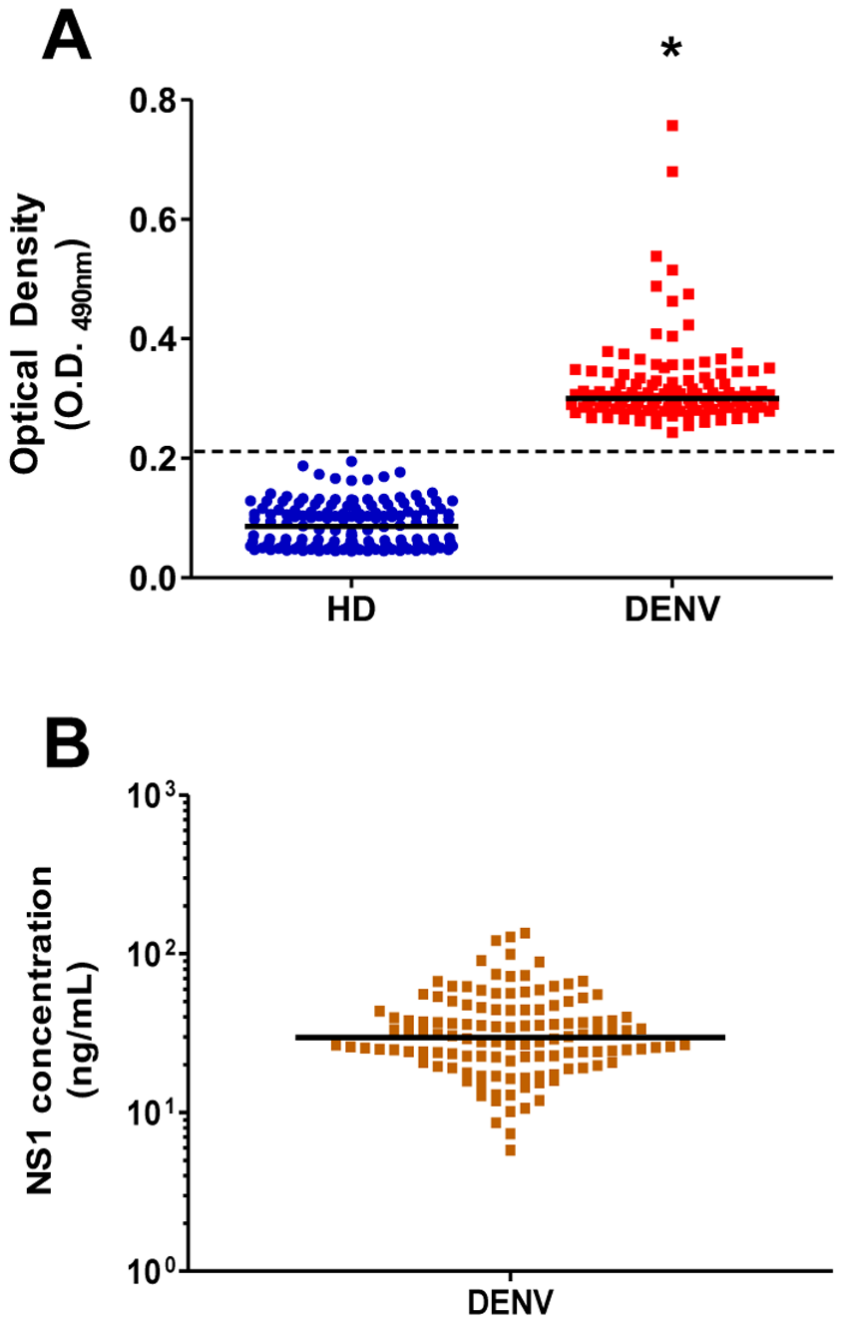
Development of a lab-based ELISA to detect NS1 antigen from the four serotypes of DENV-positive samples. Protein G-purified anti-rNS1 polyclonal antibodies from DENV2 and DENV4 were used as the capture (produced in mice) and detector (produced in rabbits) antibodies for the capture ELISA. Validation of this methodology was performed using purified rNS1 protein from the four serotypes and serotyped serum specimens (data not shown). (A) Determination of the optical density (O.D_490nm_) values from the 128 samples used in this work (DENV) and from 145 negative samples from apparently healthy blood donors (HD). (B) A standard curve was established using purified rNS1 proteins from the four DENV serotypes, and the O.D._490nm_ values were converted to NS1 concentrations in ng/mL. A *p* value less than 0.05 was considered statistically significant. *p<0.001.

First, we developed a lab-based ELISA that could similarly detect the levels of NS1 antigen from the four DENV serotypes. To achieve maximum accuracy without false-positive results, this assay was performed using different combinations of anti-NS1 antibodies for all DENV serotypes. The best result was achieved when anti-NS1 antibodies from DENV2 and DENV4 were combined for both steps of the sandwich procedure. This assay was validated with the use of purified recombinant NS1 protein from the four DENV serotypes and using previously serotyped samples from DENV-infected patients, which were different from those used in this work. It is worth noting that similar results were obtained when serum samples were diluted 1∶2 (as recommended by commercial kits, ex. Platelia Dengue NS1 Ag assay) or diluted 1∶5 (condition used in this work) (data not shown). To assess the potential for this methodology to detect NS1 antigen from Brazilian samples, all dengue-positive samples and 145 samples from apparently healthy donors (dengue-negative samples) were subjected to this ELISA. The median optical density (O.D_490nm_) value for dengue-positive samples was approximately 3-fold higher than that observed for healthy donors (Fig. 1A). The cut-off line was calculated as the mean value of dengue-negative samples plus three standard deviations. As expected, all dengue-positive samples were above the cut-off (Fig. 1A), which corresponded to a 100% positivity (p<0.001). To determine the NS1 concentration, we established a standard curve using purified rNS1; the circulating NS1 concentration in these samples varied from 7 to 284 ng/mL (Fig. 1B).

Next, NS1 positivity was evaluated using the Platelia Dengue NS1 Ag assay. We observed that the NS1 detection rate in secondary cases was clearly lower than that in primary cases (55.7% vs. 79.1%, respectively) (Table 3), which corroborates literature data. This difference was likely due to the formation of NS1: anti-NS1 complexes that impair NS1 detection. The overall NS1 positivity was similar between the dengue and dengue with warning signs groups (71.1% and 71.4%, respectively), although this rate in the severe dengue group decreased to 30%. Interestingly, the overall NS1 detection using the Platelia assay was just 68% (87/128), which is a relatively low rate of detection (Table 3). The lowest detection limit of NS1 by Platelia assay and by our lab-based ELISA was 11.94 ng/mL and 7.3 ng/mL, respectively (data not shown).

**Table 3:** Determination of NS1 positivity using Platelia Dengue NS1 Ag assay.

	No. of NS1-positivity/Total no. of samples (%)			
	Dengue	Dengue with warning signs	Severe Dengue	Total
Primary infection	39/51 (76.4)	12/13 (92.3)	2/3 (66.7)	53/67 (79.1)
Secondary infection	30/46 (65.2)	3/8 (37.5)	1/7 (14.2)	34/61 (55.7)
**Total**	69/97 (71.1)	15/21 (71.4)	3/10 (30)	87/128 (68)

As previously reported in the literature, the Platelia Dengue NS1 Ag assay fails to detect some DENV infections, mainly DENV2 and DENV4 [2], [8], [23], [24]. Based on this observation, we next sought to identify the infecting DENV serotype(s) of our samples. A serotype-specific PCR was used to detect the presence of the DENV genome in 54 samples, and these results revealed the co-existence of DENV1 (9/54) and DENV4 (45/54) infection during the 2012 dengue outbreak in Rio de Janeiro (Table 4). It is worth noting that the remaining samples were positive for NS1 (according to Platelia) and/or DENV-specific IgM/IgG assays. All DENV1 infections corresponded to mild cases of the disease (dengue without warning signs), although DENV4 was detected in samples from patients exhibiting dengue (31/40), dengue with warning signs (11/11) and severe dengue (3/3). Thus, the NS1 positivity was compared according to the infecting DENV serotype and disease severity. All DENV1-positive samples were also NS1-positive (100%), whereas only 86.7% of DENV4-positive samples were NS1-positive according to the Platelia Dengue NS1 Ag assay (Table 4). Interestingly, although 90.3% and 100% of DENV4 samples from the dengue and dengue with warning signs groups, respectively, were positive for NS1, no sample from the severe dengue group was positive for NS1 (Table 4). These results corroborate the poor detection of NS1 from DENV4 infections using the Platelia Dengue NS1 Ag assay, as reported by previous studies [2], [8], [24].

**Table 4:** Determination of the infecting serotype according to dengue case classification.

	Total		Dengue		Dengue with warning signs		Severe Dengue	
	Total (%)	Platelia positive (%)	Total (%)	Platelia positive (%)	Total (%)	Platelia positive (%)	Total (%)	Platelia positive (%)
DENV 1	9/54 (16.7)	9/9 (100)	9/40 (22.5)	9/9 (100)	0	—	0	—
DENV 4	45/54 (83.3)	39/45 (86.7)	31/40 (77.5)	28/31 (90.3)	11/11 (100)	11/11 (100)	3/3 (100)	0/3 (0)

### Determination of NS1 antigenemia

To explore the relationship between circulating NS1 levels and risk factors for the development of severe dengue, the NS1 concentration was compared with the number of days after the onset of symptoms, the presence of IgM antibodies, gender and age (Table 5). As established in the literature, the NS1 level peaked between days 3 to 5 (considering day 0 as the first day) but remained detectable until day 9 in some cases [4]. Unexpectedly, we did not observe significant differences in NS1 levels throughout the course of infection. The median NS1 concentration varied from 22.6 ng/mL on day 2 to 36.8 ng/mL on day 7 (Table 5). We did not observe a Gaussian distribution in NS1 levels according to days of fever, which might be due to the difference between informed and real first day of symptoms. Similarly, no significant difference in NS1 concentration was observed between IgM-negative and IgM-positive samples, although the median concentration was slightly higher in IgM-negative compared to IgM-positive samples (36.0 and 27.5 ng/ml, respectively - Table 5). In addition, no significant differences were observed between male and female genders (Table 5) and between different ages (Table 5).

**Table 5:** Assessing NS1 concentration in DENV-positive samples.

NS1 concentration, median (range) in ng/mL	
	Total (n = 128)
**Age (years)**	
0–10	27.9 (10.7–88.6)
11–20	30.1 (7.3–241.7)
21–50	32.4 (5.8–284.8)
>50	26.8 (15.4–64.6)
**Gender**	
Male	30.1 (10.7–135.1)
Female	31.0 (5.8–127.8)
**Day of illness**	
Day 2	22.6 (5.8–62.2)
Day 3	26.2 (7.3–62.6)
Day 4	32.3 (16.9–59.4)
Day 5	22.0 (10.1–55.3)
Day 6	28.5 (14.4–90.8)
Day 7	36.8 (24.9–73.2)
**Presence of IgM**	
IgM (+)	27.5 (10.1–90.8)
IgM (-)	36.0 (5.8–99.2)
**Serological status**	
Primary	28.5 (5.9–127.9)
Secondary	30.4 (10.2–135.1)

**Figure 2:**
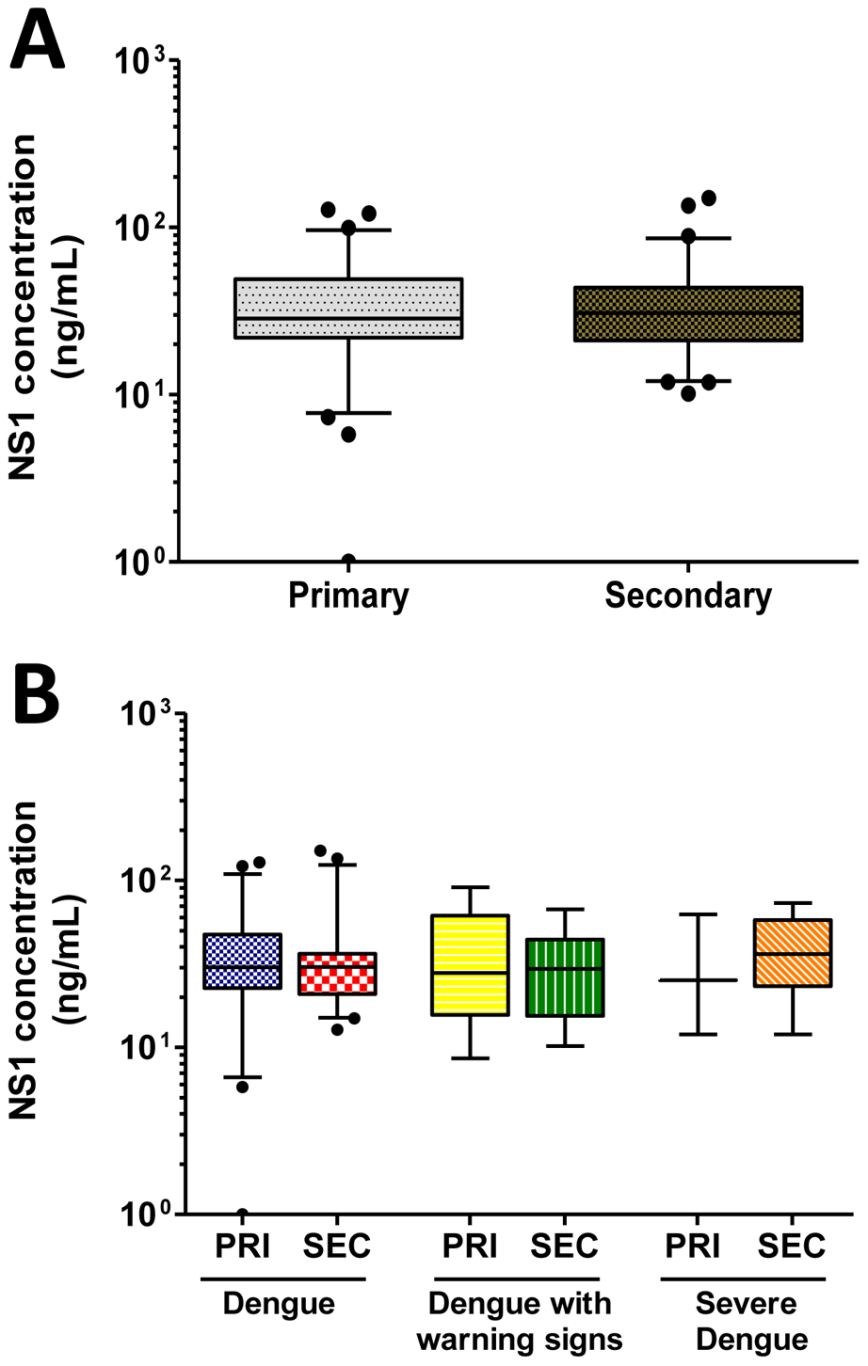
Determination of the circulating NS1 concentration according to serological status. (A) The NS1 levels were determined in primary and secondary cases. (B) Primary and secondary cases were divided according to dengue classification and compared to NS1 levels. All data are presented as the median and interquartile range (5–95%). Results were considered statistically significant for *p* values less than 0.05; no comparison was statistically significant.

Next, we evaluated the circulating NS1 levels in primary and secondary cases. In contrast to that observed in a previous study [20], the median NS1 concentration was similar in primary and secondary cases (28.5 and 30.4 ng/mL, respectively - Fig. 2A and Table 5). To evaluate whether a large dispersion in the NS1 concentration occurred in the most severe cases, the primary and secondary cases were separated according to the new dengue classification protocol. Although no significant difference in NS1 concentration was observed in any group, a large dispersion in NS1 levels occurred in the mildest case of dengue (Fig. 2B). However, statistical analysis was not possible in the severe group due to the low number of patients with primary infection. Finally, we analyzed whether circulating NS1 levels varied according to the infecting serotype. After the samples were stratified according to dengue classification and infecting serotype, we observed that the NS1 levels in DENV1 infection were slightly higher than those in DENV4 infection, although this difference was not statistically significant (Table 6). The overall median NS1 concentration was 29.1 ng/mL. Altogether, these results indicate that circulating NS1 levels do not correlate with classical dengue risk factors, at least for the DENV4 infections evaluated in this study.

**Table 6:** Assessing NS1 concentration according to Dengue case classification and infecting serotype.

	NS1 concentration, median (range) in ng/mL			
	Dengue	Dengue with warning signs	Severe Dengue	Total
DENV 1	33.3 (22.7–127.8)	—	—	33.3 (22.7–127.8)
DENV 4	29.2 (7.3–88.6)	26.7 (8.6–74.4)	36.1 (11.9–56.3)	28.0 (7.3–88.6)
**Total**	30.7 (7.3–127.8)	26.7 (8.6–74.4)	36.1 (11.9–56.3)	29.1 (7.3–127.8)

## Discussion

Despite the efforts of the WHO and the scientific community to combat recurrent dengue epidemics, this disease remains a threat to hundreds of thousands of people annually. Moreover, although millions of dollars are invested each year to develop an efficient tetravalent vaccine, this strategy has been proven to be much more difficult and complex than was initially thought. Many laboratories at reference centers are also actively searching for drugs that block viral replication, in an attempt to reduce viral infection and consequently the likelihood of developing severe cases, although this approach is far from applicable. Meanwhile, the pursuit of viral markers for early screening of disease progression has become the most feasible strategy to prevent severe and fatal cases. Because previous studies suggested that the NS1 protein may serve as a marker of disease severity [17], [18], [20], we sought to investigate the NS1 antigenemia in the scenario of an epidemic, using non-prospective samples from patients in different cities of the state of Rio de Janeiro, Brazil, who were infected during the 2012 dengue outbreak.

Considering that the clinical symptoms of hemorrhagic dengue have changed over the years, the previous guidelines for dengue classification are no longer adequate. Therefore, in 2009, the WHO revised the dengue classification guide [3]; this new protocol is essentially based on symptomatic manifestations to facilitate physician classification of patients according to the grade of severity and determine the correct clinical management. A recent study demonstrated that this guide is more adequate than the traditional scheme, albeit less specific [25]. Accordingly, we adopted this new guideline to classify the samples used in this work. It is important to mention that the criteria used to include a sample in our panel required positive results to at least one of the following tests: serotype-specific PCR, NS1 detection by the Platelia Dengue NS1 Ag assay and dengue-specific IgM/IgG assay. As expected, the majority of cases were classified as dengue without warning signs, and just 10 patients were classified as severe dengue, including three patients who succumbed to the disease. Although Brazil is the most affected country in the Americas, recurrent DENV epidemics are recent, as confirmed by the fact that the majority of patients were adults, with a median age of 26 years. Another interesting observation was that 52.3% were primary cases, reflecting that there is yet a remaining population naïve to dengue infection.

Previous studies have evaluated the sensitivity of early dengue diagnostic assays based on the detection of NS1 protein. Undoubtedly, NS1 detection represents the best approach for acute-phase diagnosis when compared to viremia levels, as NS1 can be detected throughout the febrile phase [4] and is more stable in solution than the viral genome, thus facilitating sample handling and storage. Notwithstanding, the sensitivity of commercial kits varies according to the patients' immune status and infecting serotype, with lower levels of detection reported in patients experiencing secondary infection and/or infection by DENV2 or DENV4 [2], [7], [8], [23], [24], [26]. In this context, we developed a lab-based ELISA that could detect the NS1 antigen from all four DENV serotypes at a high level of sensitivity (data not shown). Using this methodology, we were able to detect the NS1 antigen in all evaluated samples, reflecting a positivity rate of 100%. It is also important to mention that we achieved these results using less amount of samples (1∶5 dilution) than that recommended by Platelia assay (1∶2 dilution). We did not observe any differences in sensitivity between primary and secondary cases, most likely because our polyclonal antibodies recognized the NS1 antigen as strongly as those raised during a secondary infection. Not surprisingly, the Platelia assay showed a lower sensitivity to secondary cases when compared to primary ones. However, the overall low sensitivity of the Platelia assay was unexpected (just 68%). As a result, we further analyzed which DENV serotypes were circulating during the outbreak studied and found that at least two serotypes, DENV1 and DENV4, co-circulated. Together, our results are in accordance with those of previous studies, revealing the low sensitivity of the Platelia assay to DENV4 infection.

The major obstacle for treating dengue is to predict, early during infection, which individuals are susceptible to severe cases. In a pioneer prospective study in Thailand, DENV2-infected children experiencing DHF showed an increase in circulating NS1 concentration during the first 72 h of infection, suggesting that NS1 protein may be used as a viral marker for disease severity [17], and the same finding was observed in another study also conducted in Thailand [18]. Conversely, a study performed in Vietnam reported that the NS1 concentration was significantly higher in DENV1 and DENV3 infections than in DENV2 infection, and no correlation was observed between NS1 levels and disease severity [27]. A further study conducted in Mexico suggested that secreted NS1 concentrations were higher in patients with dengue hemorrhagic fever experiencing primary infection than in those with DF, although the level depended on the infecting serotype and patients' immune status [20].

Taking these results into account, we calculated the circulating NS1 levels in our samples and found that this level ranged from 7 to 284 ng/mL. Interestingly, the lowest NS1 level detected by Platelia assay was 11.94 ng/mL, which was higher than that achieved in our system (7.3 ng/mL). We did not observe any significant difference in NS1 concentration among all conditions analyzed. However, any conclusion based on these results should be done with caution. First, the decision to seek medical intervention was made by the patients, and the information about days of fever was provided by them, which may not correspond precisely to the beginning of illness. Second, after initial screening, the samples from individuals with probable dengue were collected and sent to LACEN-RJ to confirm DENV infection using serological assays and perform epidemiological bulletins. No patient was enrolled in a prospective study; therefore, we do not have any information about the evolution of the patient, except for those presenting severe cases. Altogether, the lack of precise information and paired samples limit any hypothesis about the relationship of NS1 levels and dengue severity, however it does not rule out the conclusion that the concentration range of NS1 during DENV4 infection is lower than those previously described in the literature [4], [17], [18], [20]. In conclusion, our work corroborates the findings of previous studies and underscores the low efficiency of the Platelia assay to detect NS1 antigen from DENV4 infection. In addition, we developed a lab-based ELISA that can similarly detect NS1 protein from the four DENV serotypes, and this assay showed 100% positivity. Using this approach, we were able to calculate the circulating NS1 concentration in retrospective samples from a 2012 dengue outbreak that occurred in Rio de Janeiro, Brazil. It is worth noting that this is the first study to perform a ‘field-analysis’ of NS1 antigenemia in terms of the circulating concentration using Brazilian samples and the new WHO guideline for dengue classification.
